# The mental health of working women after the COVID-19 pandemic: an assessment of the effect of the rise in sexual harassment during the pandemic on the mental health of Pakistani women using DASS-21

**DOI:** 10.3389/fpsyt.2023.1119932

**Published:** 2023-07-14

**Authors:** Shehzeen Akbar, Pasha Ghazal

**Affiliations:** Department of Biosciences, COMSATS University Islamabad, Islamabad, Pakistan

**Keywords:** sexual harassment, working women, DASS-21, COVID-19 pandemic, mental disorders among Pakistani women

## Abstract

**Introduction:**

The mental health of South Asian women has been observed to be in regression lately, with sexual harassment as one of the major factors accounting for mental health deterioration, especially for women who leave their homes frequently for work and study. The COVID-19 pandemic not only augmented the mental health distress of the general female population but the rise in sexual violence against women is being consistently reported around the globe. Based on this background, we adopted a two-pronged strategy to assess whether working women and students aged 18–55 experienced a rise in sexual harassment in the 18 months after lifting the COVID-19 lockdowns. Secondly, using the well-validated psychometric test, DASS-21, we evaluated the psychiatric outcome of this change on the mental health of those women.

**Study design:**

The study was designed as a quantitative, cross-sectional survey-based research.

**Methodology:**

A total of 303 women participated in this study. Personal interviews through a specifically designed questionnaire and psychometric test DASS-21 were administered to assess the mental health state of working women and female students, aged between 18 and 55 years old. The mean age of the participants was 37 ± 2.8. The study population was further categorized into two main groups of limited and frequent interactions based on varying levels of the frequency of leaving home and interacting with male strangers in their daily routine. Data were analyzed and the correlation between limited/frequent interaction and DASS-21 total scores and sub-scores of depression, anxiety and stress, and other sociodemographic variables were investigated using the Chi-square test, whereas psychosocial predictors of mental distress were evaluated using multiple linear regression analysis after matching limited and frequent interaction groups using a 1:1 propensity score-matched pair method for sociodemographic covariates.

**Results:**

Overall, approximately 50% of our study population experienced changes in the behavior of male strangers that could be categorized as harassment in their daily life interactions, whereas 33.66% of participants experienced relatively more sexual harassment post-pandemic than before it. This observation was significantly correlated with the frequency of male interaction (*χ*2 = 5.71, *p* < 0.01). Overall, 34% of our study population scored >60 on the DASS21-total score, whereas 29.04% scored >21 on the depression scale. Alarmingly, >40% of the women in the frequent interaction group scored in the extremely severe range of anxiety and depression. Moreover, in the regression analysis, out of all the factors analyzed, the extent of everyday interaction with male strangers, an increase in fear of sexual crimes, and a self-perceived increase in mental distress during the 18 months post-pandemic were found to be highly statistically significant predictors of mental distress not only for total DASS 21 but also for the sub-scales of depression, anxiety, and stress.

**Conclusion:**

In Pakistan, women experienced a rise in sexual harassment cases post–COVID–19. An increase in sexual harassment was found to be a predictor of negative mental health in the form of depression, anxiety, and stress.

## Introduction

Sexual harassment is one of the major causes of mental health deterioration among women, specifically among those who leave home every day for work and study and have frequent interactions with male strangers (1, 2). It is a very prevalent crime against women worldwide (3). In Pakistan, sexual harassment is the most prevalent form of gender-based violence (4). The local emergency helpline (Madadgaar) reported that approximately 93% of women in Pakistan have experienced some sort of sexual harassment in their lives (5).

An excessive prevalence of sexual harassment in the country brings about a state of regressive mental health conditions among its victims. Continuous exposure to the same stressor and underreporting because of social stigma and possibly due to the fear of restriction placements on mobility for education or work often lead to the incubation of these symptoms over time, which causes the development of mania, psychosis, aggressive behavior, and ultimately suicidal thoughts (6). Unfortunately, Pakistan is one of those South Asian countries where women experience a two-to three-fold increased incidence of psychiatric disorders, compared to men (7). Moreover, they also form a major group in the country who attempt non-suicidal self-injurious behavior (NSSIB) (8). The Baluchistan-based study showed that 69.3% of victims of sexual harassment reported extremely severe depression, 97.1% reported extremely severe anxiety, and 79% reported severe and extremely severe stress levels (9).

Other than Pakistan, Bhutan, Bangladesh, India, Nepal, Srilanka, and Maldives are a few other South Asian countries where sexual harassment in public spaces has been normalized and is termed indirectly as “eve-teasing.” Women have learned to live with the “normality of unsafety” (10, 11). In Bangladesh, sexual harassment in public transport is rampant to the extent that according to many women, traveling in public transport is like “going to war”; women are compelled to weigh their economic prosperity against the actual risk to their security. Consequently, increased incidences of sexual harassment are limiting women’s participation in the labor force which in turn directly impacts the economic growth of the country (12). In India alone, sexual harassment has become extremely pervasive during the last 20 years, with rape being reported as the fourth most common crime in India (13). Alarmingly, despite an exponential rise in crime rates, only a very slight percentage of the victims (fewer than 1.5%) will ever report to the police (14). Although, in recent years, a slight improvement in reporting has been observed following a very high-profile fatal gang rape and murder in Delhi in a moving public bus which sent shock waves across the region (15). Ironically, all the countries of the South Asian region have enacted laws against sexual harassment; nevertheless, their enforcement remains a far bigger challenge. It is a grim reality that victims of sexual assault, usually women or children, often choose not to register a complaint out of fear of intense victimization and the biased attitudes of service providers. Therefore, very few women are courageous enough to report incidents of sexual violence to the police; most endure these unscrupulous acts silently (16). In Pakistan, in light of the recent rise in harassment incidents and underreporting, the government has sought help from digital media to increase reporting. Now, victims can report cases of assaults *via* a “police web portal” but can also track progress on their cases online (17). The e-portal can be quite helpful for city dwellers and tech-savvy individuals, but still, reporting such crimes in rural areas and slums poses a challenge to the government. Despite the overall improvement in the reporting of sexual assaults, conviction rates are quite low across the region, ranging from 2 to 3% in Bangladesh to 4% in India which reflects the level of impunity the perpetrators enjoy in the South Asian region despite strong projections of such cases in electronic and print media. The existing justice system in these countries is heavily plagued by corruption at all hierarchical levels. Consequently, this acts as a major impediment to accessing justice for the survivors. Across all six South Asian countries, pressure is put on the survivor or her family to withdraw the complaint and enter into an extra-legal settlement with the perpetrator. In Bangladesh, India, and Nepal, over 60% of the survivors interviewed reported facing pressure to settle/compromise the case. Lack of protection from retaliation or further threats coupled with weak judicial procedures opens the door to out-of-court settlements that force victims to drop charges, reinforcing a climate of impunity for perpetrators (18). In Pakistan, according to law, rape is a non-compoundable offense; nevertheless, the conviction rates are quite low (2–3%) which have been slightly improved to 16% with the establishment of special courts for gender-based violence working under the recent anti-rape act 2021 (19). Still, protection gaps in the law, biased attitudes of the service providers, delays in medical examinations, and prosecution and trials by the police are major barriers to accessing justice coupled with the risk of ostracization of the victim by society.

It is a grim reality that pandemics tend to intensify the persecution of women which often leads to a rise in domestic and sexual violence. Rates of sexual violence increase during states of emergency, including natural disasters, wars, and health crises. This could possibly be because of an increase in enabling environments for gender violence to occur or due to an exacerbation of underlying drivers of violence against women and girls such as a failure of law enforcement and an increase in gender inequalities and unequal social norms (20).

The COVID-19 pandemic not only intensified the mental health distress of the general female population by widening the social and economic disparities but consistent reports of a rise in sexual violence against women also augmented the mental health burden further. In Pakistan, the health impact of COVID-19 has not been as devastating as feared, possibly due to the younger demographics of the country. Nevertheless, the pandemic exposed and exacerbated some of the country’s major weaknesses such as poverty, gender inequality, and persecution. During the pandemic (since January 2020), a rise in gender-based violence was expected. The United Nations predicted a 20% increase in both physical and sexual violence during the COVID-19 pandemic (21) with 15 million more cases for every 3 months of lockdowns (22). The reality perfectly aligned with the expectations, especially in South Asia; multiple reports of increased gender-based violence in the region were made (23–26). Specifically, in Pakistan, during the first to the third month of 2020, violence against women increased by 200% and rape, specifically, by 300% although it is believed that many more cases exist but have not been reported (27). However, despite an apparent increase in the number of cases of sexual harassment, no such studies have been reported so far on the subject from Pakistan.

Therefore, in this first-of-its-kind study, we sought to assess: 1) if working women and female students of various socioeconomic backgrounds and professions had experienced increased sexual harassment during the past 18 months after lifting the COVID-19 lockdowns, 2) observed changes in the behavior of male strangers that can fall under the definition of sexual harassment, 3) the resulting impact on the mental health of these women based on the findings of a recent empirical study that observed that men are usually the main perpetrators of sexual harassment crimes for both men and women (28), 4) We also tried to analyze if having more male interactions in daily life meant more frequent experiences of sexual harassment, and 5) We also investigated whether the frequency of leaving home and the extent of interaction with male strangers could be a predictor of mental distress in our study population.

In our study, we adopted the tripartite model of sexual harassment proposed by Gelfand et al. (29). According to this model, sexual harassment can be grouped into three types of actions: gender harassment, unwanted sexual attention, and sexual coercion or *quid pro quo* sexual harassment. Gender harassment refers to all forms and extents of demeaning and intimidating verbal and non-verbal acts and behaviors toward women; these can range from taunts to flashing genitalia or sexual images. Unwanted sexual attention includes verbal and non-verbal acts of a sexual nature that invade someone’s personal space such as making sexual remarks aimed at someone, touching them, or attempting a sexual assault. Sexual coercion means threatening or demanding sexual favors in response to benefits - specifically job-related benefits. Overall, sexual harassment covers a wide range of behaviors and actions ranging from staring, catcalling, groping, and gesturing inappropriately, to sending inappropriate sexual text messages, assaults, attempted rapes and rapes (29, 30).

### Methodology

#### Study population

**Figure 1:**
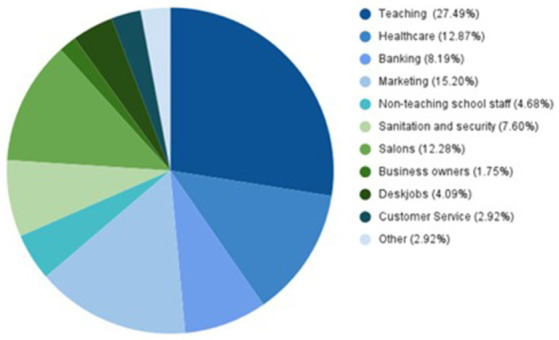
Occupational industries of working women.

This multi-site, cross-sectional study was conducted among 303 women aged between 18 and 55 years who belonged to different socio-economic classes. The study was conducted for 3 months from September 2021 to November 2021 in many different cities all over Pakistan. In these cities, personal visits were made to sites such as banks, salons, hospitals, shopping malls, cash-and-carry stores, pharmacies, clinics, private and government schools, colleges, and other educational institutions like academies, universities, and vocational training centers (see Figure 1). This ensured a broad range of professional backgrounds of the study population ranging from students and teachers to nurses and sanitation workers and even women security guards with varying levels of frequency of leaving home and interaction with male strangers in their daily life routine. The participants were individually approached and asked for their consent to participate in the research survey. The participants were briefed about the objectives of the study and the tripartite model of sexual harassment was explained to them. The option to leave the survey anytime during the data collection was given to all the respondents. The inclusion criteria of the study comprised working women and students aged 18 to 55 years old, with or without a past diagnosis of depression, stress, and/or anxiety, with varying degrees of frequency of leaving home for work or study; they were defined as the study population. The study excluded women below 18 or older than 55 years of age. Patients with a medical history of mental diseases such as dementia, Alzheimer’s, Schizophrenia, and psychosis were also excluded from the present study to minimize confounding due to neuropsychiatric disorders, and the study of the effects of sexual harassment on patients of the aforementioned disorders was out of scope of the present study.

The study was carried out in adherence to the Helsinki Declaration of 1975 as revised in 2000, and the research study was accepted and approved by the Ethics Review Board of the Department of Biosciences, COMSATS University, Islamabad, and informed consent was obtained from all the participants.

#### Study instruments

This was a multi-site, multi-city study in which personal visits were made to various locations and cities to ensure participation by a wide range of professions and sociodemographic backgrounds (see Figure 1). The two-part survey was conducted by a team of trained female interviewers (psychologists), who approached women in different settings and comprehensively explained the purpose of the survey and the tripartite model of sexual harassment. The context period in which information was sought, i.e., 18 months after the COVID-19 pandemic lockdowns were lifted, was clearly mentioned and then the willingness to participate was sought. All the participants who showed a willingness to participate were requested to provide written informed consent for recruitment. The interviewers conducted the first part of the survey, whereas since DASS-21 is a self-reported questionnaire, it was filled out by the participants. The interviews were conducted in a comfortable setting at the participant’s workplace, hostel, or university in case of students.

The questionnaire consisted of three parts. The first part of the questionnaire included questions related to demographics: participants’ age, level of education, nativity, household income, and relationship status. In addition to this, specific questions were asked about participants’ commute and work routines and levels of male interaction. The second part comprised questions specifically related to sexual harassment experiences in the past 18 months after the lifting of the COVID-19 lockdown in Pakistan. Participants were asked if they perceived any changes in behavior or interactions (which can be categorized as sexual/verbal/physical harassment) with male strangers in their routine encounters before and after the pandemic/COVID-19, e.g., the interviewers explained the behaviors to them and asked if they had observed an increase in the occurrence of more men catcalling, making lewd gestures, touching inappropriately, or women experiencing increased incidences of rape in accordance with the definition of sexual harassment. They were also asked if they have experienced more sexual harassment after the COVID-19 pandemic than before it. In addition to this, participants were asked whether their fear of sexual harassment-related crimes has increased in the past 18 months/ after the COVID-19 pandemic and in what ways this has affected their lifestyle or everyday routine. The third part of the questionnaire was related to their mental health status. Participants were asked if they felt increasingly stressed /anxious or depressed in recent times. Additionally, they were asked about the reasons that greatly contributed to their increase in anxiety/stress and depression or worsened already existing depression symptoms. The frequency or the exact number of times a participant experienced sexual harassment was not included in a deliberate attempt to increase study participation and maintain confidentiality.

In addition, the current mental health status of all the participants was assessed using the established psychometric test, DASS-21. The participants filled out the questionnaire themselves. Additional information regarding opinions and experiences of sexual harassment and mental health, as well as past diagnoses of mental health, was also obtained from the participants through the interviews.

### DASS-21

The Depression, Anxiety, and Stress Scale (DASS– 21) has 21 questions that cover a wide range of symptoms of depression, anxiety, and stress (see DASS -21 questionnaire in Supplementary files). Each of the questions was stated in the first person, assertive tone, and required the participants to grade on a 4-point scale their experiences with a score of 0 (did not apply to me at all), 1 (applied to me to some degree or some of the time), 2 (applied to me to a considerable degree or a good part of the time), and 3 (applied to me very much or most of the time).

In recent years, accumulating data suggest good reliability, construct, and structure validity of DASS -21 in the Pakistani Population (31–33). Therefore, we found this test suitable for the assessment of psychometric measures in Pakistani working women. In our study population, we assessed the construct reliability using Cronbach’s alpha. Overall, for our study population, DASS 21 had αn alpha value of 0.939 for 21 items which showed good reliability. High construct reliability was also obtained for clinical sub-categories. Depression had an alpha value of 0.846, anxiety had an alpha value of 0.828, and stress had an alpha value of 0.843. Concerning validity, in Pearson’s correlation test, all study questions were significantly correlated at 0.01 level (two-tailed).

After the DASS-21 data collection, for analysis, the scores for depression, anxiety, and stress were separated, added, and each subcategory was multiplied by two. The score range for each of the three categories was 0 to 42. The scores were then measured against the scales provided for each of the sub-categories, categorizing participants into groups of normal, mild, moderate, severe, and extremely severe. Total DASS-21 scores were also calculated (see Supplementary information). The total scores were the cumulative addition of all sub-category scores. Cut-off scores of 60 and 21 were used, respectively, for the total DASS score and the depression subscale. These cut-off scores were derived from a set of severity ratings proposed by Lovibond and Lovibond (34). Scores ≥60 (for DASS-total) and ≥ 21 (for the depression subscale) are labeled as “high” or “severe.” For those with total scores of 60 or over, professional psychiatric consultation was advised.

We defined the participants as Normal, Mild, Moderate, Severe, and Extremely Severe for each of the three sub-categories of DASS-21, i.e., Depression, Anxiety, and Stress using the scale originally defined by Lovibond and Lovibond (34). The DASS-21 test is based on the assumption that the differences between depression, anxiety, and stress experienced by normal individuals and clinical populations are different in degree. In this study, the scores, therefore, do not point toward objective disorders but rather provide insights into the overall population severity levels. For depression, 0–9 scores formulated the range for normal levels of depression in the population, 10–13 referred to mild rates of depression, 14–20 pointed toward moderate levels of depression severity, 21–27 represented severe levels, and scores over 28 pointed toward extremely severe depression rates. For the DASS-21 subscale of anxiety, 0–7 scores referred to normal population levels. Having a score of 8 or 9 for anxiety questions translated to mild anxiety, 10–14 were scores for moderate levels, and 15–19 were for severe levels of anxiety. A score of 20 was defined as the highest cut-off score and all individuals with 20+ scores showed strong anxiety levels in the population. For the DASS-21 subsection of stress, 0–14 were only for individuals with normal levels, 15–18 were mild levels, and 26–33 were for severe levels of stress. For stress, the highest cut-off score was 34, and scores above that referred to extremely severe levels of stress.

### Statistical tests

Quantitative values were described using their numbers, mean, frequency, percentage, and standard deviation. For categorical variables, we performed a Chi-square test of independence to analyze the association of the extent of male interaction (limited and frequent) being taken as the dependent factor with all the categorical variables. For continuous variables and assessment of predictors of psychological conditions, a linear regression test was applied with DASS-21 total score and individual scores of depression, stress, and anxiety taken as dependent variables after matching with propensity score analysis. We used a 1:1 propensity score-matched pair method combined with covariate adjustment to analyze both limited and frequent interaction groups. The unbalanced conditions at baseline between the two groups were controlled by using PS matching with covariate adjustment. The 1:1 PS matching yielded 95 pairs of matched subjects between limited and frequent interaction groups, resulting in no differences in age, education, income, profession, locality, or relationship status. Data sets were analyzed using the statistical software SPSS version 25.

## Results

### General characteristics of the study population

#### Demographic groups

Our study population comprised of 303 participants who originated from various socioeconomic backgrounds and professions (see Figure 1). All the participants were women; the majority of them were young (67.66%) in the 18 to 35 years age group, whereas 32.34% were middle-aged between 36 to 55 years. The mean age of the participants was 37 ± 2.8. The majority (55.78%) were students, whereas 19.47% had post-graduate level education. Concerning nativity, 16.5% of women belonged to rural regions and 83.5% were from urban areas. In terms of socio-economic background, the majority of the study population (59.08%) belonged to the middle-class economic background with a monthly household income of 50,000 to 200,000 PKR. In our study population, 49.5% of the women were single and 50.49% were in committed relationships. By profession, the two major groups of participants were either students who formed 43.56% of the study population, or working women who formed 56.44% of the total population. For the commute, the majority of the participants (50.17%) walked to and from their educational institutions/workplaces; 41.58% were frequent users of busses; 28.71% used taxis, and 25.74% used ride-hailing services. A good majority of the total study population (49.17%) used other modes of commute as well; these modes of commute included self-driving, passage with family, or company-provided commute (see Table 1).

**Table 1:** Extent of male interaction in participants’ daily lives and its association with various categorical study variables.

		Total (*n* = 303)	Limited interaction *N* = 136	Frequent interaction *N* = 167	*χ* ^2^	*p*-value
Age	18–35	205 (67.66%)	73 (53.68%)	132 (79.04%)	22.04	0.00001
	36–55	98 (32.34%)	63 (46.32%)	35 (20.96%)		
Level of education	≤12 years	75 (24.75%)	42 (30.88%)	33 (19.76%)	6.98	0.03
	Bachelors	169 (55.78%)	65 (47.79%)	104 (62.28%)		
	Post-graduate	59 (19.47%)	29 (21.32%)	30 (17.96%)		
Nativity	Rural	50 (16.5%)	23 (16.91%)	27 (16.17%)	0.03	>0.05
	Urban	253 (83.5%)	113 (83.09%)	140 (83.83%)		
Household income range	Less than 50 k	73 (24.09%)	35 (25.74%)	38 (22.75%)	7.54	0.02
	50 k – 200 k	179 (59.08%)	87 (63.97%)	92 (55.09%)		
	More than 200 k	51 (16.83%)	14 (10.3%)	37 (22.16%)		
Relationship status	Single	150 (49.51%)	53 (38.97%)	97 (58.08%)	10.95	0.0009
	Committed	153 (50.49%)	83 (61.03%)	70 (41.92%)		
Profession	Student	132 (43.56%)	47 (34.56%)	85 (50.9%)	8.14	0.0043
	Working woman	171 (56.44%)	89 (65.44%)	82 (49.10%)		
Frequency of leaving home	Everyday	235 (77.56%)	100 (73.53%)	135 (80.84%)	2.39	>0.05
	Often, on a weekly basis	52 (17.16%)	27 (19.85%)	25 (14.97%)		
	Occasionally	16 (5.28%)	09 (6.62%)	07 (4.19%)		
Mode of commute	Walk	152 (50.17%)	62 (45.59%)	90 (53.89%)	10.441	0.034
	Bus	126 (41.58%)	39 (28.68%)	87 (52.1%)		
	Taxi	87 (28.71%)	30 (22.06%)	57 (34.13%)		
	Ride hailing services	78 (25.74%)	22 (16.18%)	56 (33.53%)		
	Others^1^	149 (49.17%)	68 (50%)	81 (48.50%)		
Perceived changes in behavior 18 months post- COVID-19	Yes	149 (49.17%)	55 (40.44%)	94 (56.29%)	7.53	0.006
	No	154 (50.83%)	81 (59.56%)	73 (43.71%)		
Experienced more SH 18 months post-COVID-19?	Yes	102 (33.66%)	36 (26.47%)	66 (39.52%)	5.71	0.01
	No	201 (66.34%)	100 (73.53%)	101 (60.48%)		
Increased fear of crime during pandemic period	Yes	213 (70.3%)	82 (60.29%)	131 (78.44%)	11.82	0.0005
	No	90 (29.70%)	54 (39.71%)	36 (21.56%)		
Effect of increased fear of crime 18 months post-COVID-19	Protective attitudes^2^	203 (67%)	80 (58.82%)	123 (73.65%)	53.95	0.00001
	Defensive attitudes^3^	119 (39.27%)	18 (13.24%)	101 (60.48%)		
	Vigilant attitudes^4^	120 (39.60%)	29 (21.32%)	91 (54.49%)		
	Others^5^	07 (2.31%)	03 (2.21%)	04 (2.4%)		
	Fear of crime did not increase	90 (29.70%)	54 (39.71%)	36 (21.56%)		
Feel increasingly stressed, anxious, depressed in recent times	Yes	242 (79.87%)	100 (73.53%)	142 (85.03%)	6.17	0.01
	No	61 (20.13%)	36 (26.47%)	25 (14.97%)		
Reasons behind feeling more stressed, anxious, depressed in recent times	Increased fear of incidence of SH during the COVID-19 pandemic	142 (46.86%)	43 (31.62%)	99 (59.28%)	15.43	0.001
	pandemic related work/study stress	170 (56.11%)	69 (50.74%)	101 (60.48%)		
	Domestic violence	41 (13.53%)	19 (13.97%)	22 (13.17%)		
	Do not feel more depressed, stress, or anxious	61 (20.13%)	36 (26.47%)	25 (14.97%)		

*χ*^2^ values show relationship between limited and frequent male interaction groups, SH, sexual harassment. 1 = Includes self-driving, traveling with family, using booked vans or company provided commute. 2 = Includes sharing tracking link/car number with friends or family during commute, avoiding going out alone, avoiding going out at night. 3 = Includes carrying weapons or pepper sprays, wearing more culturally accepted clothing. 4 = Includes checking for secret cameras in public try-rooms, and restrooms, avoiding sharing contact information (phone number, address) with a strangers, avoiding interacting with men that women expresses their distrust in 5 = For example excessive worry for related younger women as their reaction to increase in fear of crime during the COVID-19 period, frequent change of cell numbers to avoid identification.

##### Frequency of leaving home and sexual harassment

One of the important variables for this study was to assess whether the probability of sexual harassment has any association with the frequency of leaving home (every day vs. often) and the extent of interaction with male strangers (limited vs. frequent). Our data showed that 77.56% of the total study population was comprised of individuals who leave their homes every day. Out of these, 73.53% of participants had frequent interactions with male strangers. Meanwhile, in the limited interaction group, this proportion was lower (44.88%). Of the remaining participants, a smaller proportion of 17.16% leaves often or weekly, whereas a small group of 5.28% leaves their homes only occasionally.

##### Sexual harassment experiences post-COVID-19 pandemic

Overall, 49.17% of our population affirmed that they had perceived changes in the behavior of male strangers that could be categorized as harassment in their daily life interactions, after the lifting of lockdowns, post-COVID-19 pandemic. This observation was highly significantly related to the frequency of interaction with male strangers (*χ*^2^ = 7.53, *p* < 0.006); of the participants with frequent interactions, 56.29% noticed violating male behavior. Whereas, intriguingly, a good majority (59.56%) in the limited interaction group did not perceive such changes. Of note, overall, 33.66% of our study population reported experiencing relatively more sexual harassment, post-COVID-19 pandemic than before it. An increased occurrence of incidents of sexual harassment during COVID-19 was significantly correlated with the frequency of male interaction (*χ*^2^ = 5.71, *p* < 0.01). Looking at population data, we see that a larger proportion of participants (39.52%) with frequent male interaction experienced increased sexual harassment in the post-pandemic period, whereas comparatively much less (26.47%) reported such negative behavior within the limited male interaction group. In our study population, the extent of male interaction was more frequent in women below the age of 35, effectively increasing their chances of sexual harassment; therefore, 18–35 years old participants reported almost a two-to-three-fold increase in experiences of sexual harassment both generally and specifically during the COVID-19 period in comparison to participants aged 36–55 years. Similar trends were observed for single women, as well as for students at the undergraduate level of education having the highest fear of sexual crime, followed by those with 12 or fewer years of education, which was followed by participants at the postgraduate level. Concerning economic classes, the middle class was found to have the maximum male interaction. In our study, we observed those with incomes below 50,000 had limited male interactions. We assume that this might be because these participants were in jobs that had gender-segregated workplaces, for example, female-only salons and sanitation workers often working in the all-female set-up such as girls’ schools and colleges.

##### Frequency of interaction with male strangers and fear of sexual harassment

Women interacting frequently with men were found to harbor an increased fear of sexual harassment in comparison to those with lesser male interactions (*χ*^2^ = 11.82, *p <* 0.0005). Overall, 70.3% reported an increased fear of the crime of sexual harassment during COVID-19. Among women with frequent male interactions, 78.44% reported an increase in fear of crime. Whereas, this proportion was less (60.29%) in the limited interaction group. An escalation of the fear of becoming a victim of sexual harassment during COVID-19 heightened the sense of insecurity among women and brought about behavioral changes of a protective and defensive nature (see Table 1). Among those in the frequent interaction group, 73.65% became more protective, 54.49% more vigilant, 60.48% more defensive, and 2.4% took up other behaviors such as having different contact numbers for work and personal communication. Intriguingly, the magnitude of this behavioral change was not lesser in the limited interaction group; 58.82% reported taking up protective behaviors, 13.24% adopted defensive attitudes, 21.32% started being more vigilant, and 2.21% took up other preventive measures by staying in continuous contact whenever traveling alone or feeling unsafe. This adoption of preventive attitudes in response to increased fear of crime was found to be significantly associated with the frequency of interaction with male strangers (*χ*^2^ = 53.95, *p* < 0.00001).

Overall, a large majority of study participants (79.87%) reported being increasingly mentally distressed during the COVID-19 pandemic, which, in line with the earlier observed trend, the frequent interaction group formed a relatively larger proportion (85.03%) than the limited interaction group (73.53%). This overall increase in stress, anxiety, and/or depression post-COVID-19 period was significantly related to the extent of male interaction in daily life (*χ*^2^ = 6.17, *p <* 0.01).

The reasons behind increased self-perceived stress, anxiety, and/or depression were highly significantly related to everyday male interaction (*χ*^2^ = 15.43, *p* < 0.001). The stark difference between the limited and frequent male interaction groups for reasons for being depressed, anxious, or stressed was “increased fear of the incidence of sexual harassment during the COVID-19 pandemic,” with 59.28% from the frequent male interaction group feeling increasingly distressed because of this reason, whereas only 31.62% of those with limited male interaction opted for this reason for their mental distress. Work and study burden due to pandemic-related factors were the second most frequent response, with 60.48% belonging to the frequent male interaction group vs. 50.74% prevalence in the limited interaction group. The option of domestic violence was opted for by almost an equal number of respondents in the limited (13.97%) and frequent (13.17%) male interaction groups.

Overall, in the DASS-21 questionnaire, on average, our study population scored below the 60-cut-off range for total DASS-21 scores (48.97 ± 1.69), whereas a good proportion (34%) of the study population scored >60, and the rest of the participants (66.01%) scored <60. Student’s t-test showed a statistically significant difference in the total DASS- 21 scores of those who scored ≤60 vs. those who scored ≥60 [t (1,301) =23.29, *p* < 0.0001], which highlights the fact that in our study population, there were at least 103 (34%) participants who scored ≥60; hence, psychiatric/clinical consultation was advised for these participants (35). The majority of the participants scored in the normal range of the three sub-categories of DASS-21; however, for anxiety, the population mean was 15.66 ± 0.59, which fell in the severe range. Notably, in relation to this, a greater majority (36.63%) had scores in the extremely severe range for anxiety (Table 2).

**Table 2:** Chi-square analysis of the relationship between extent of male interaction and DASS-21 scores.

DASS-21 classification	Total participants *N* = 303	Limited interaction group	Mean DASS-21 scores	Frequent interaction group	Mean DASS-21 scores	χ^2^	*p*-value
*Depression* *Mean ± SD*	15.47 ± 0.62	N = 136 (44.8%)	12.24 ± 0.79	N = 167 (55.11%)	18.12 ± 0.87	22.01	<0.05
Normal (0–9)	96 (31.68%)	55 (40.44%)	3.49 ± 0.40	41 (24.55%)	4.2 ± 0.48		
Mild (10–13)	38 (12.54%)	21 (15.44%)	11.14 ± 0.22	17 (10.18%)	10.94 ± 0.25		
Moderate (14–20)	81 (26.73%)	38 (27.94%)	16.3 ± 0.29	43 (25.75%)	17 ± 0.34		
Severe (21–27)	40 (13.20%)	11 (8.09%)	23.63 ± 1.28	29 (17.37%)	23.7 ± 0.28		
Extreme severe 28+	48 (15.84%)	11 (8.09%)	32.3 ± 1.07	37 (22.16%)	34 ± 0.93		
DASS score (<21)	215 (70.96%)	114 (83.82%)	9.92 ± 0.43	101 (60.48%)	10.73 ± 0.63	19.82	<0.0001
DASS score (>21)	88 (29.04%)	22 (16.18%)	28 ± 1.10	66 (39.52%)	29.4 ± 0.82		
*Anxiety* *Mean ± SD*	15.66 ± 0.59	N = 136 (44.8%)	12.29 ± 0.80	N = 167(55.11%)	18.41 ± 0.79	28.68	<0.0001
Normal (0–7)	71 (23.43%)	45 (33.09%)	3 ± 0.35	26 (15.57%)	3.23 ± 0.49		
Mild (8–9)	23 (7.59%)	14 (10.3%)	8 ± 0	9 (5.39%)	8 ± 0		
Moderate (10–14)	60 (19.80%)	32 (23.53%)	12.75 ± 0.26	28 (16.77%)	12.35 ± 0.27		
Severe (15–19)	38 (12.54%)	15 (11.03%)	16.93 ± 0.26	23 (13.77%)	16.60 ± 0.20		
Extreme severe (20+)	111 (36.63%)	30 (22.06%)	26.2 ± 1.06	81 (48.50%)	27.03 ± 0.68		
*Stress* *Mean ± SD*	17.84 ± 0.60	N = 136(44.8%)	14.74 ± 0.82	N = 167 (55.11%)	20.44 ± 0.81	16.429	<0.002
Normal (0–14)	132 (43.56%)	74 (54.41%)	7.7 ± 1.31	58(34.73%)	9.97 ± 0.94		
Mild (15–18)	38 (12.54%)	17 (12.5%)	16.35 ± 0.19	21 (12.57%)	17.23 ± 0.21		
Moderate (19–25)	62 (20.46%)	26 (19.12%)	21.46 ± 0.34	36 (21.56%)	22 ± 0.28		
Severe (26–33)	46 (15.18%)	13 (9.56%)	27.69 ± 0.54	33 (19.76%)	28.06 ± 0.38		
Extreme severe (34+)	25 (8.25%)	6 (4.41%)	37.33 ± 1.11	19 (11.38%)	38.3 ± 0.74		
*Total DASS-21scores* *Mean ± SD*	48.97 ± 1.69	N = 136 (44.8%)	39.26 ± 2.24	N = 167(55.11%)	59.96 ± 2.28	21.98	<0.0001
≥ 60	103 (33.99%)	27 (19.85%)	80.46 ± 2.78	76 (45.51%)	83.8 ± 2.13		
< 60	200 (66.01%)	109 (80.15%)	29.52 ± 1.64	91 (54.49%)	36.54 ± 1.8		

Data represents mean (±SD) scores and % of participants in each subcategory. Chi-square analysis was performed on the % of participants in the respective sub-categories (*p* < 0.05).

#### The extent of male interaction and DASS-21 results

The study population mean scores for total DASS-21 and sub-categories were found to vary greatly with the extent of interaction with male strangers; as a result, stark differences in mean DASS-21scores for total and clinical sub-categories (depression, anxiety, and stress) were observed between the limited and frequent interaction groups. Participants in the frequent interaction group had a mean total score of 60 ± 2.28, which is an alarming observation and reflective of the distressed psychological state of women who had relatively increased encounters with male strangers in daily routines; in contrast, this score was found to be many folds less and was in the normal range (below 60) for participants in the limited interaction group (39.26 ± 2.24). Significantly, more numbers of participants in the frequent group had scores in the >60 range (45.5% vs.19.8%). The chi-square test revealed a highly significant inter-group association [*χ*^2^ = 21.98, *p* < 0.0001] (see Table 2).

The DASS-21 subscale scores for depression, stress, and anxiety showed linear trends of increase with the frequency of male interaction in everyday life. The extent of male interaction showed the strongest trends for anxiety, implying that women with increased everyday interactions with men had higher levels of anxiety. Anxiety levels were found to be highly significantly different between our two variable groups and among those with frequent daily life interaction with men; 48.50% fell into the extremely severe range of anxiety, whereas in the contrasting group with reduced male interaction, approximately half (22.06%) had a score in that range [χ2 = 195.2, *p* < 0.0001]. Similarly, a significantly greater number of participants had scores in the severe (17.37%) and extremely severe range (22.16%) of depression in the frequent interaction group [*χ*^2^ = 22.01, *p* < 0.05]. Of note, twice the number 39.52% of the women in the frequent interaction group had ≥21 depression scores, a cutoff value for identifying extremely severe depression (35) [*χ*^2^ = 19.82, *p* < 0.0001]. For DASS-21 stress subscales, in the frequent interaction group, 19.76% were in severe and 11.38% were in the extremely severe stress group, whereas among those with limited interaction with men, only 9.56% of participants had scores in the severe subscale and only 4.41% participants had scores in the extremely severe stress sub-scale. The two groups had an overall highly significant intergroup association [*χ*^2^ = 16.429, *p* < 0.002] (see Table 2).

Overall, the level of mental distress was found to be much higher in women who had frequent interaction with male strangers every day versus those who had limited interactions.

The psychological impact of the increase in sexual harassment crimes during COVID-19 and its association with the two clinical cut-offs (>60 and < 60) for total DASS-21 scores were evaluated to understand the mental health conditions of women who leave home every day for study or work (see Table 3 for details). Overall, in all perceived behaviors, a greater percentage of participants who responded in the affirmative had scores in the above-normal range, i.e., >60 than those who did not. This observation was statistically significant and the chi-square test showed a strong association with the response variable and the DASS-21 clinical cut-off ranges of the total DASS-21 scale (see Table 3). From the analysis, it was evident that a significantly greater percentage (40%) of those participants who responded in the affirmative were likely to score in the severe range ≥ 60 in total DASS-21 than those who did not. Of participants with total DASS-21 scores of 60 or above, 49% responded in the affirmative of experiencing increased changes in violating male behavior in their routine interactions post-COVID-19 pandemic. Of this proportion, 46.31% scored above 60 while 53.69% scored below 60. A relatively small proportion (22.08%) did not report any such observations or experiences. Whereas 33.66% responded in the affirmative for experiencing more sexual harassment after the pandemic than before it; out of this, 49.02% scored >60 whereas 51% scored <60. Intriguingly, 73% of the participants who responded negatively to this question scored <60 in total DASS 21 scores. A large majority of our study population (70.3%) reported an increase in fear of sexual harassment crimes in the past 18 months after the lifting of COVID-19 lockdowns; of these, 42.25% scored >60 on the total DASS 21 score. Whereas out of 29.07% who did not perceive any increase in the fear of the crime, 85.56% scored <60 of the total DASS-21 scores. Approximately 79.87% of our study population reported feeling increasingly stressed, anxious, or depressed in recent times after the COVID-19 pandemic. Of these, 39.97% scored above 60 in total DASS21 scores, whereas only 20.13% of participants did not agree with the statement and scored <60 in total DASS21 scores.

**Table 3:** Chi-Square table for analysis of association of DASS-21 total scores cutoff values >60 and < 60 with sexual harassment and related behavioral changes before and after COVID-19.

Variable questions	DASS-21 total scores	Number of participants with positive response to questions *N* (%) (mean DASS-21 scores)	Number of participants with negative response to questions *N* (%) (meanDASS-21 scores)	*χ* ^2^	*p*-value
Do you perceive any changes, in behaviors (which can be categorized as sexual / verbal /physical harassment), with male strangers in your routine encounters, before and after the pandemic/COVID-19?		*N* = 149 (49.17%)	*N* = 154(50.83%)	19.81	<0.00001
	≥ 60	69 (46.31%)	34 (22.08%)		
	≤ 60	80 (53.69%)	120 (77.92%)		
Do you think you have experienced more sexual harassment after the COVID-19 pandemic, than before it?		*N* = 102 (33.66%)	*N* = 201 (66.34%)	15.47	<0.00008
	≥ 60	50 (49.02%)	53 (26.37%)		
	≤ 60	52 (50.98%)	148 (73.63%)		
Has your fear of crime increased in the past 18th months/ after Covid − 19 pandemic?		*N* = 213 (70.3%)	*N* = 90 (29.70%)	21.8	<0.00001
	≥ 60	90 (42.25%)	13 (14.44%)		
	≤ 60	123 (57.75%)	77 (85.56%)		
Do you feel increasingly stressed /anxious or depressed, in recent times		*N* = 242 (79.87%)	*N* = 61 (20.13%)	29.5	<0.00001
	≥ 60	96 (39.97%)	2 (3.28%)		
	≤ 60	146 (60.33%)	59 (96.72%)		

SH, sexual harassment.

#### Factors acting as predictors of mental distress

To identify psychosocial factors which could act as predictors of mental distress, we used multiple regression models with a total DASS 21 score and the clinical sub-categories as dependent variables. To control for confounding by demographic variables (age, education, profession, income, locality, and relationship status), we performed a 1:1 propensity score matching between the limited and frequent interaction groups which yielded 190 matched subjects. The demographic variables were not found to be significantly different after matching. Whereas out of all the psychosocial factors analyzed, the extent of everyday interaction with male strangers, the effects of an increase in fear of sexual crimes, and self-perceived increase in mental distress during the 18 months post-pandemic were found to be highly statistically significant predictors of mental distress not only for total DASS-21 (see Table 4) but also for all the clinical sub-categories. Intriguingly, for the subcategory of anxiety, the frequency of leaving home was also found to be significant. We observed that an increase or decrease in everyday life male interaction was significantly related to an increase in DASS-21 total score, depression scores, anxiety scores, and stress scores. The predictor variable “effects of an increase in the fear of the crime of sexual harassment" was strong enough to induce behavioral and decisional changes in the study participants and was found to be highly significantly associated with an increase in DASS-21 total scores and all sub-categories. Our study participants reported an increase in mental distress due to three main reasons: an increase in work/study stress, domestic violence, or an increase in fear of crime or sexual harassment in the first 18 months of the pandemic period. Of these, fear of sexual harassment was highly significantly associated with an increase in psychometric levels of depression, anxiety, and stress and was reported by 46.86% of the participants.

**Table 4:** Multiple linear regression model for DASS-21 total scores, depression, anxiety, and stress and independent variables on, 190 matched subjects with 1:1 propensity score matching between limited and frequent male interaction groups, for covariates (age, education, income, profession, locality, relationship status).

Variables	DASS-Total				DASS-Depression				DASS-Anxiety				DASS-Stress			
	B-value	*p*-value	Lower bound (95%)	Upper bound (95%)	B-value	*p*-value	Lower bound (95%)	Upper bound (95%)	B-value	*p*-value	Lower bound (95%)	Upper bound (95%)	B-value	*p*-value	Lower bound (95%)	Upper bound (95%)
Male Interaction	10.309	**0.004**	17.332	3.286	3.904	**0.005**	6.597	1.211	3.179	**0.015**	5.728	0.630	3.226	**0.017**	5.862	0.590
Age	3.813	0.466	14.125	6.500	1.000	0.618	4.954	2.954	1.373	0.470	5.116	2.369	1.429	0.467	5.301	2.442
Education	9.493	0.107	2.079	21.065	3.959	0.081	0.489	8.408	3.579	0.094	0.621	7.778	2.070	0.348	2.274	6.414
Locality	5.102	0.267	3.948	14.151	1.332	0.450	2.141	4.804	2.183	0.191	1.101	5.467	1.536	0.373	1.861	4.933
Income	0.583	0.929	13.422	12.256	1.140	0.649	6.076	3.796	0.568	0.810	4.092	5.227	0.140	0.954	4.960	4.680
Relationship status	0.911	0.834	7.653	9.475	0.806	0.629	2.479	4.090	0.592	0.708	3.700	2.517	0.728	0.655	2.487	3.943
Profession	9.011	0.083	1.188	19.209	3.947	0.48	0.035	7.859	3.442	0.068	0.259	7.144	1.660	0.393	2.169	5.489
F of Leaving Home	11.703	0.132	3.578	26.984	2.235	0.454	3.641	8.111	7.810	**0.006**	2.265	13.356	1.816	0.533	3.920	7.553
Behavior post-	1.708	0.714	10.903	7.488	1.284	0.473	2.242	4.809	0.649	0.702	3.986	2.688	2.346	0.181	5.798	1.105
↑ SH after COVID	5.446	0.272	15.192	4.301	3.104	0.104	6.848	0.639	0.720	0.688	4.257	2.818	1.541	0.407	5.200	2.118
Fear of crime	23.095	0.326	23.225	69.415	7.893	0.382	9.869	25.656	10.975	0.199	5.836	27.785	4.344	0.623	13.045	21.732
Effects of ↑ FoC	16.260	**0.001**	25.343	7.177	4.266	**0.017**	7.749	0.783	5.051	**0.003**	8.347	1.754	6.928	**<0.0001**	10.338	3.518
Increase in DAS	25.924	**<0.0001**	34.390	17.458	9.159	**<0.0001**	12.405	5.912	8.082	**<0.0001**	11.155	5.010	8.705	**<0.0001**	11.883	5.527

F of leaving home; Frequency of leaving home; FoC, fear of crime; SH, Sexual Harassment. Bold values meaning *p* < 0.05.

## Discussion

Pandemics usually have inequitable effects on the most vulnerable groups of society. The COVID-19 pandemic was no exception. Although the causality rate was twice higher for men than women, the pandemic has affected women more not only socially and economically by increasing the load of unpaid care work and precarious employment situations but also, foremost, by intensifying gender-based violence against women and girls exponentially (36). The statistical data released by the United Nations (37) for gender-based violence around the world is alarming and shows an unprecedented increase in domestic and sexual violence, unequivocally, in both third-world developing countries as well as in developed nations, e.g., domestic violence in France increased by 30 percent following the country’s lockdown on March 17, whereas during the first 2 weeks of lockdown in Spain, the emergency number for domestic violence received 18 percent more calls, and the helplines in Singapore have received 30 percent more calls. However, research on the impact of gender-based violence on the mental health of its victims is still scarce, especially among women.

In the aftermath of the COVID-19 pandemic and an associated rise in gender-based violence, the UN (38) specifically recommended making endeavors to collect more sex-disaggregated data to analyze the impact of pandemics on women and address the increased demands.

To the best of our knowledge, this is the first study from Pakistan that quantitatively investigated the psychometric effects of the rise in sexual harassment, specifically during the COVID-19 pandemic in a cohort of working women and the resulting change in their mental health and behavior. In this study, we made efforts to collect sex-disaggregated data on the increase in sexual violence among Pakistani women who leave home every day for work or study. We specifically tried to target those women who are hard to reach and were not vocal about their rights such as sanitation workers, female security guards, nurses, and office receptionists. Such data is central to the much-needed policy change, specifically in a developing country like Pakistan where sexual and domestic violence is a prevalent crime (4); COVID-19 further intensified the problem. According to the policy brief released by the Ministry of Human right on the “gendered impact of COVID-19 in Pakistan,” 28% of women aged between 15 and 49 years old have experienced physical violence whereas 6% have experienced sexual violence, and nearly 7% of the women who have ever been pregnant have experienced violence during their pregnancy and 34% of ever-married women have experienced spousal physical, sexual, or emotional violence during the pandemic period (39). Corroborating these reports, our study explicitly showed that during the COVID-19 pandemic, working women and students experienced a rise in sexual harassment, which was found to be affecting their mental health not only in their perception but also in psychometric measures, as was reflected by the DASS-21 results. Of our study population, 34% scored >60 in total DASS -21 scores and 29% scored >21 in the depression subscale, a cutoff value at which clinical intervention is suggested to be sought according to Beaufort et al. (35). Another important observation of the study within the span of the last 2 years of the COVID-19 pandemic was that while half of our study population (49.17%) reported increased sexual harassment and men’s inappropriate behaviors, alarmingly, a greater proportion (70.3%) reported heightened worry and fear of becoming the victim of sexual harassment. The majority in our study were frequent users of public transport and reported a rise in experiences of sexual harassment in public spaces as well as sexual abuse inside homes.

We also discovered that the increase in sexual harassment during COVID-19 was highly significantly related to depression, anxiety, and stress. Experiencing traumatic events can understandably cause mental distress; however, an important finding was that the heightened fear of sexual harassment among women was strong enough to influence their mental state and predispose them to the development of depression, stress, and anxiety.

Of note, an increased frequency of interaction with male strangers in daily life was one of the stronger predictors of depression, anxiety, and stress in women, together with the effects of fear of becoming a victim of sexual harassment and self-perceptions of increased mental distress. Interestingly, in our study, these factors continued to be significant predictors irrespective of age, locality, education level, socio-economic, and marital status, which reflects the degree of pervasiveness of sexual harassment in our society. During lockdowns, even though male interaction was likely to be reduced, many women were still working online as well as in-person in industries like healthcare. Moreover, the complete lockdown in Pakistan lasted only for a few months and was lifted in May 2020 (40), and many industries started to get back to normal routines. This meant increased male interaction for most working women. These can be the reason that led to an increased fear of crime and escalated mental distress to the extent that the majority of our study population demonstrated behaviors that limited their personal and social life. The more frequently women interacted with men, the more they tend to adopt defensive behaviors. To avoid sexual harassment, the most prevalent behavior was to avoid going out alone and at night. Many started to wear more culturally acceptable clothing, which would range from simple *shalwar kameez* (a type of suit worn especially by Asian women, with long shirts and loose trousers worn together with long scarfs) (dupatta /chaddars) and *abayas* (full-length gowns) for different people. Usually, non-traditional or provocative clothing is believed to lead to sexual objectification and, hence, biases the perception of sexual violence. It is common for people to increase the extent of victim blaming and letting go of the abusers’ crimes after judging the victims’ attire (41). This is precisely what explains donning more clothing as an act of defense as after incidents of harassment, clothes are often a major part of the discussion, and a woman in non-traditional attire might be blamed for luring the harasser. On the contrary, studies have shown that convicted harassers and rapists do not remember the attire of their victims (42); therefore, the main reason sexual assaults take place is because a perpetrator committed that offense, irrespective of the victims’ attire. In fact, culture is central to the theory of sexual objectification, an observation first made by Fredrickson and Roberts in 1997 (43). According to this theory, bodies exist within social and cultural contexts and are viewed in light of the prevalent socio-cultural practices in that society.

In Pakistan, women are judged by their physical appearance and are considered the source of physical attraction, which prevents many women from comfortably leaving their homes; ironically, in the view of their male counterparts, it’s the responsibility of “women to cover themselves and stay inside their homes.”

Sexual objectification has more adverse consequences for women than men (44–46), affecting mental health, and intellectual performance, and increasing the risk of depression (47, 48). Objectification also tends to make women behave as lesser beings in social interactions (45) which in turn produces profound effects on the victim’s mental health (49).

Studies have shown that sexual harassment and the fear of becoming a victim are chronic stressors that may put victimized workers under severe mental and physical stress and compromise their mental health to a large extent, which if left undiagnosed and untreated, might gradually lead to the development of post-traumatic stress disorders, suicidal ideations, eating disorders, or phobic or somatoform disorders (50, 51). Unfortunately, in South Asia, due to strong patriarchal mindsets, women from puberty have learned to endure their mental distress as part of life and pretend to act normal than to seek help. Moreover, across the South Asian region and particularly in Pakistan, the subject of mental health treatment is associated with stigma, far greater for women than men, which prevents individuals from seeking help, in turn raising the toll of undiagnosed cases. Our study underscores the ever-increasing demand for setting up psychosocial support for working women because a lack of adequate domestic and emotional support can compromise mental health further. Moreover, across the South Asian region, governments need to comprehensively reform the justice system by addressing protection gaps in the sexual harassment laws, improving service providers’ response in cases of sexual violence, and overall improvement of prosecution procedures and trials of sexual offenses to effectively combat the enormity of sexual harassment.

### Study limitations

Our study hosts a few limitations that can be addressed in future research. Foremost, sexual harassment is a taboo issue in Pakistan and women are often reticent about narrating their experiences; some participants might have underreported their experiences, specifically the number of times they had been a victim of sexual harassment. For this reason, we could not investigate the numerical frequency of sexual harassment before and during COVID-19. During interviews, some participants’ hesitation was evident showing that they did not feel comfortable sharing their experiences explicitly. However, we covered it to a greater extent by the breadth of qualitative questions on the topic. This study was also limited by time constraints and, hence, only 303 participants could be recruited. A larger sampling size could provide better insight into the state of women’s mental health. Another limitation was that social desirability bias may affect participants’ responses to the scale of anxiety, and they might have exaggerated in expressing their level of anxiety due to the overall COVID-19 scenario. Therefore, to minimize this kind of bias, participants were requested to state their actual feelings and perceptions due to which we included close-ended questions together with open-ended ones. However, this might have led to response bias and participants might have felt constrained in their answers. Additionally, collecting data through a self-administered questionnaire carries risks for selection bias although we tried to reduce the selection bias by recruiting participants from a vast number of professions and socio-demographic backgrounds. Nevertheless, the cross-sectional design obscured the true mental state of our participants since we had to rely on subjective reports rather than screening the respondents for psychopathologies according to a known disease classification system.

## Conclusion

Research studies highlighting the impact of COVID-19 on mental health issues are scarce, especially in South Asian women. This research study has opened avenues for understanding the mental health consequences of increasing levels of sexual harassment during and after pandemics. Our study also supports the feasibility and ease of use of DASS-21 as a simple tool to identify individuals who might be prone to develop psychopathologies and are suffering from mental distress. Now, the far bigger challenge for the present governments and organizations is to recognize that the COVID-19 pandemic is affecting the psychosocial well-being of men and women differently; therefore, there is a dire need of creating gender-sensitive intervention programs and policies following effective responses to mental health challenges in the wake of pandemics like COVID-19. To achieve this goal, the UN recommends allocating additional resources to protect women, putting women at the center of policy changes, and making endeavors to collect more sex-disaggregated data to analyze the impact of pandemics on women (38).

## Data availability statement

The original contributions presented in the study are included in the article/Supplementary material, further inquiries can be directed to the corresponding author.

## Ethics statement

The studies involving human participants were reviewed and approved by Ethics Review Board, Department of Biosciences, COMSATS University Islamabad. The patients/participants provided their written informed consent to participate in this study.

## Author contributions

SA performed data curation and helped in manuscript writing. PG designed and supervised the study, performed the statistical analysis, and wrote the manuscript. All authors contributed to the article and approved the submitted version.

## Conflict of interest

The authors declare that the research was conducted in the absence of any commercial or financial relationships that could be construed as a potential conflict of interest.

## Publisher’s note

All claims expressed in this article are solely those of the authors and do not necessarily represent those of their affiliated organizations, or those of the publisher, the editors and the reviewers. Any product that may be evaluated in this article, or claim that may be made by its manufacturer, is not guaranteed or endorsed by the publisher.
