# Supplementary material for: The mental health of working women after the COVID-19 pandemic: an assessment of the effect of the rise in sexual harassment during the pandemic on the mental health of Pakistani women using DASS-21
Source: Front Psychiatry. 2023 Jul 14;14:1119932. doi: 10.3389/fpsyt.2023.1119932 (PMC10382200; doi:10.3389/fpsyt.2023.1119932)
Supplement: Supplementary file 1 [file Data_Sheet_1.pdf]

Questionnaire for assessment of mental health conditions of women during the COVID-19 Pandemic in Pakistan

Demographics:

1. Please select the age range you fall in.
O 18-35
O 35-55

2. What is your level of education? (Check one that applies best)
O Below 12 years
O Bachelors
O Post-graduate

3. Which one applies best to your locality of residence?
O Rural
O Urban

4. Name of City/District?

---

5. What is your income range (PKR)?

O Less than 50k per month
O 50k – 200k per month
O More than 200k per month

6. What is your relationship status?
O Single
O Committed

Work, Commute:

7. Which one describes you better?
O Student
O Working woman
O Both

8. How often do you leave your home?
O Everyday
O Often, on a weekly basis
O Occasionally

9. What is the extent of male interaction in your job, and during commute?
O Frequent
O Limited

10. Which one of these do you often use as your mode of commute, especially to and from workplace or educational institution? (Check as many as applicable)
□ Walk
□ Bus
□ Taxi
□ Ride hailing services
□ Others (__________)

11. Do you worry about sexual harassment before/during commute when travelling alone with stranger(s)?
O Yes
O No

COVID-19 and behavioral changes
12. Do you perceive any changes, in behaviors or interactions (which can be categorized as sexual /
verbal /physical harassment), with male colleagues, driver, fruit vendor, or any other male strangers in your routine encounters, before and after the pandemic/COVID-19?
O Yes
O No

13. Do you think you have experienced more sexual harassment after the COVID-19 pandemic, than
before it?
O Yes
O No

14. Has your fear of crime increased in the past 18th months/ after Covid -19 pandemic?
O Yes
O No

15. If yes, in what way this fear has affected your lifestyle / everyday routine? (Check as many as
applicable)

□ Sharing tracking link/car number with friends or family during commute
□ Carrying a weapon or pepper spray
□ Wearing more culturally accepted clothing
□ Avoid going out alone
□ Avoid going out at night
□ Check for secret cameras in public try-rooms, and restrooms
□ Avoid sharing your contact information (phone number, address) with a stranger
□ Avoid interacting with a man you know if a woman expresses their distrust in them
□ Ohers (______)

Mental health
16. Do you feel increasingly stressed /anxious or depressed, in recent times?
O Yes

O No

17. In your view which of these factors, has greatly contributed to increase in anxiety/stress and
depression or worsen already existing depression symptoms. (Check as many as applicable)
□ Increased fear of incidence of sexual harassment in times of COVID -19 pandemic
□ Pandemic related work /study stress
□ Domestic violence
□ Other (_________)

The End
